# The Popularity of "The Services."

**Published:** 1912-07-13

**Authors:** 


### THE POPULARITY OF "THE SERVICES."

In a modest single-page appendix to the minutes of the proceedings of the General Medical Council is printed a report by the Examination Committee of that body on the entrants into the Medical Services of the Crown. This report discloses a state of affairs which cannot be called satisfactory, for it reveals that for 119 vacancies in the Naval Medical Service, Army Medical Service, and Indian Medical Service during the year there were but 137 candidates. A few years ago various steps were taken to increase the popularity of these important Services, and for a time competition to enter them was quite brisk. But it seems as if somehow this popularity has waned, which is not only undesirable in itself, but is also detrimental to the efficiency of the armed forces of the nation. The Navy still maintains a certain measure of popularity; for twenty-one vacancies there were thirty-seven candidates, so a good choice was available. But the Royal Army Medical Corps was compelled to accept every single one of the seventy-one candidates who applied, and the once-favourite Indian Medical Service had but twenty-nine men among whom to award twenty-seven commissions.

It is thus evident that the land forces are failing dismally to attract towards their Medical Services a sufficient number of young doctors; and it is inevitable that with competition so restricted a number of men who are properly ineligible must obtain admission. What the causes of this serious state of affairs may be it should be the duty of the War Office and the India Office to ascertain without delay. When these causes, which now threaten to interfere gravely with the efficiency of the Services, have been discovered every possible means of removing them should be adopted. To judge by the trend of general opinion in Anglo-Indian circles, one of the root-causes most detrimental to the attractiveness of the Indian Medical Service is the policy in high quarters of encouraging the influx of native Indians into its commissioned ranks. Whether this is or is not the case, the Secretary of State for India may well have his attention drawn to the most disquieting evidences of lost popularity which the figures we have quoted afford.

Of the Naval Medical Service there is less need for investigation, for the proportion of candidates to vacancies is still high enough. But the Royal Army Medical Corps is in a bad way, for it is clear that it is failing to attract anything like the number of would-be entrants to ensure a sufficiently lofty standard of professional and military attainments. Within the last ten years the character and standing of the men entering this Service have been in general excellent, for the failing popularity of the Indian Service brought many recruits of the right sort to the British Service. It is evident, therefore, that these officers are, in the main, dissatisfied with their positions and prospects, and are advising their friends to avoid the same fate. It must be the care of the War Office authorities to consider very carefully the remedy for this; and having discovered it to apply it at once. They cannot and must not be allowed to remain supine in face of the present situation, but must tackle it in earnest without any delay. Indeed, for our part, we consider they have been blameworthy for allowing matters to drift to this discreditable crisis, and it is for them to redeem the confidence reposed in them as speedily as possible. Only by vigorous action can they regain the confidence of the public and the profession in regard to the Medical Services. Without such confidence there can be no question of popularity.

